# Extracellular Vesicles Released From Prostate Cancer Cells Confer Pro‐Tumor Properties to Adipocytes by Stimulating Lipolysis

**DOI:** 10.1002/biof.70067

**Published:** 2025-12-11

**Authors:** Gaia Giannitti, Kinga Kamińska, Sara Marchesi, Riccardo Garavaglia, Ivan Preosto, Małgorzata Grzesiak, Fabrizio Fontana

**Affiliations:** ^1^ Department of Pharmacological and Biomolecular Sciences “Rodolfo Paoletti” University of Milan Milan Italy; ^2^ Department of Endocrinology, Institute of Zoology and Biomedical Research, Faculty of Biology Jagiellonian University Krakow Poland; ^3^ Department of Animal Biotechnology University of Agriculture in Krakow Krakow Poland

**Keywords:** adipocytes, Akt, extracellular vesicles, free fatty acids, lipolysis, prostate cancer

## Abstract

There is consistent evidence of an association between obesity and the risk of prostate cancer (PCa). A crosstalk between PCa and adipocytes has been highlighted; however, the role of extracellular vesicles (EVs) in this communication still needs to be elucidated. Herein, we demonstrated that PCa EVs can trigger lipolysis in 3T3‐L1 adipose cells, by downregulating G0/G1 switch protein 2 (G0S2), inducing adipose triglyceride lipase (ATGL) expression and activating the cyclic AMP (cAMP)/protein kinase A (PKA)/hormone‐sensitive lipase (HSL) signaling pathway. Interestingly, we showed that the free fatty acids (FFAs) released from the EV‐treated adipocytes could increase PCa cell proliferation and clonogenic ability. Moreover, they promoted tumor cell migration and invasion, while parallelly reducing the induction of anoikis. Mechanistically, FFAs were found to trigger Akt activation, and pharmacological inhibition of this protein by BEZ235 could successfully counteract their cancer‐promoting effects. Collectively, these results support the presence of an EV‐driven bidirectional interplay between PCa cells and adipocytes, which reprograms the latter toward a lipolytic, tumor‐promoting phenotype.

## Graphical abstract

Extracellular vesicles (EVs) from prostate cancer (PCa) cells induce the release of fatty acids (FFAs) from adipocytes, which in turn sustain tumor cell proliferation and migration via Akt activation.

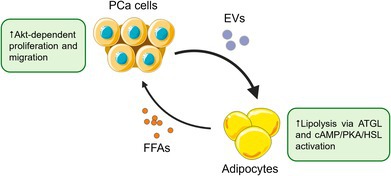

## 1 Introduction

Prostate cancer (PCa) ranks among the deadliest neoplastic diseases in men globally [1]. While primary tumors can be effectively managed through radical proctectomy, radiotherapy and androgen deprivation treatment, metastatic castration‐resistant carcinomas are still very insidious, due to their ability to escape standard approaches [2, 3, 4]. Notably, a significant rise in PCa incidence has been reported in the last years, paralleled by a remarkable increase in obesity and metabolic syndrome prevalence [5, 6, 7, 8, 9]. In particular, an obese condition has been linked to more aggressive tumors and poorer therapeutic outcomes; among the various mechanisms underlying this association, increased production of free fatty acids (FFAs) by the periprostatic adipose tissue seems to specifically drive cancer progression [5, 6, 7, 8, 9, 10].

EVs are 50–1000 nm‐sized, membrane‐bound particles that orchestrate intercellular communication through the horizontal transfer of bioactive molecules from donor to recipient cells [11]. Remarkably, they are known to mediate the interactions occurring in the tumor stroma, including those between cancer cells and adipocytes [12, 13]. Indeed, recent research has demonstrated that the vesicles released from different malignancies, such as breast, colon, liver, lung and pancreatic carcinoma, can induce pro‐tumor lipolysis in the adipose compartment, favoring its switch into a cancer‐associated phenotype characterized by reduced triglyceride storage and the release of large amounts of FFAs [14, 15, 16]. Nonetheless, it is still unclear whether these particles are involved in the adipose tissue‐driven PCa evolution.

This study aimed to further characterize the molecular interactions between PCa cells and adipocytes, focusing on the contribution of EVs to the formation of a tumor‐supporting microenvironment.

## 2 Experimental Procedures

### 2.1 Chemicals

BEZ235 was obtained from Selleckchem (Houston, TX, USA).

For Western blot analyses, the following primary antibodies were used: anti‐ALIX (2171), anti‐p‐PKA substrate (9624), anti‐HSL (4107), anti‐p‐HSL (45804), anti‐ATGL (2439), anti‐Akt (2938), anti‐p‐Akt (9271), and anti‐GAPDH (5174) from Cell Signaling Technology Inc. (Danvers, MA, USA); anti‐TSG101 (ab30871) from Abcam (Cambridge, UK); anti‐Hsc70 (13D3), anti‐calnexin (AF18) and anti‐G062 (polyclonal) from Thermo Fisher Scientific (Waltham, MA, USA); anti‐cytochrome *c* antibody (sc‐13560) from Santa Cruz Biotechnology Inc. (Santa Cruz, CA, USA). They were all used at a dilution of 1:1000.

Secondary antibodies conjugated to horseradish peroxidase (HRP) were purchased from Cell Signaling Technology Inc., while enhanced chemiluminescence (ECL) detection reagents were from Cyanagen (Bologna, Italy).

### 2.2 Cell Lines and Cell Culture

PC3 and DU145 PCa cell lines were obtained from the American Type Culture Collection (ATCC, Manassas, VA, USA) and cultured in RPMI medium supplemented with 10% fetal bovine serum (FBS), glutamine, and antibiotics. RWPE‐1 normal prostate epithelial cells were also from ATCC and grown in keratinocyte‐SFM medium supplemented with Bovine Pituitary Extract (BPE) and EGF (2.5 μM) (Thermo Fisher Scientific). 3T3‐L1 pre‐adipocytes, also sourced from ATCC, were cultured in DMEM supplemented with 10% FBS, glutamine, and antibiotics. Cells were maintained in a humidified incubator at 37°C with 5% CO_2_ and 95% air. Original cell stocks were cryopreserved in liquid nitrogen and, upon thawing, were cultured for no longer than 10–12 weeks. Cells were passaged weekly using a trypsin–EDTA solution.

### 2.3 3T3‐L1 Cell Differentiation

3T3‐L1 pre‐adipocyte differentiation was obtained as described in [17]. Briefly, it was initiated by replacing the regular culture medium with induction medium containing 10% FBS, 500 μM 3‐isobutyl‐1‐methylxanthine, 1 μM dexamethasone, 1 μg/mL insulin, and 1 μM rosiglitazone. After 3 days, the induction medium was replaced with DMEM supplemented with 10% FBS and 1 μg/mL insulin, and cells were cultured for an additional 4 days. Mature adipocytes were then maintained in regular growth medium for a further 3 days.

### 2.4 EV Isolation

EV isolation was performed as described in [18]. Briefly, FBS was ultracentrifuged at 120,000 *g* for 16 h; RPMI was then supplemented with EV‐depleted bovine serum to prepare EV‐depleted medium (EDM). PCa cells in T75 flasks at 70%–80% confluence were incubated in EDM for 48 h. EVs were isolated from 40 mL of conditioned medium by size exclusion chromatography (SEC). The medium was first centrifuged at 300 *g* for 5 min, followed by centrifugation at 16,500 *g* for 20 min at 4°C. It was then concentrated by centrifugation at 3000 *g* via a Vivaspin PES 100 kDa cutoff filter (Sigma‐Aldrich, Milano, Italy), and a 0.5 mL aliquot of this concentrate was loaded onto a sepharose‐based column (Bio‐Rad Laboratories, Hercules, CA, USA) and eluted with PBS. Fractions 7 through 11 (0.5 mL each) were pooled and further concentrated by filtration at 3000 *g* using a Vivaspin PES 5 kDa cutoff filter (Sigma‐Aldrich) to a final volume of 100 μL. Isolated EVs were stored at −80°C when not used immediately.

### 2.5 Nanoparticle Tracking Analysis

The EV size and concentration were measured using a NanoSight LM10 instrument (Malvern Instruments Ltd., Malvern, UK) equipped with a sCMOS camera (Hamamatsu Photonics, Hamamatsu, Japan) and a 450‐nm wavelength blue laser, utilizing nanoparticle tracking analysis (NTA). The obtained data were processed using NTA analytical software (version 3.1 Build 3.1.45). Particle concentration and size profiles were analyzed with the following settings: camera level at 14, shutter value of 1259 and slider gain of 366. Each measurement consisted of five 30 s movies recorded at 30 frames/s.

### 2.6 Transmission Electron Microscopy

Morphological evaluation of EVs was carried out by transmission electron microscopy (TEM). Briefly, each EV sample was fixed in 2% paraformaldehyde (Sigma Aldrich) overnight and then was deposited on carbon‐formvar coated copper 300 mesh grids (Merck Millipore, Burlington, MA, USA) for 20 min. Afterward, they were washed with PBS and transferred to 1% glutaraldehyde (Sigma Aldrich) for 5 min of fixation. Then, the samples were rinsed with distilled water and stained with uranyl oxalate for contrast enhancement (pH 7.0) for 5 min, followed by contrast and embedding in 4% uranyl acetate and 2% methyl cellulose (Chemapol, Prague, Czech Republic) on ice. Finally, the grids were air‐dried for approximately 10 min and examined using a JEOL JEM 2100HT transmission electron microscope (Jeol Ltd., Tokyo, Japan) at an accelerating voltage of 80 kV. Images were captured with a 4 k × 4 k camera (TVIPS) using EMMENU software version 4.0.9.87.

### 2.7 Measurement of Lipid Accumulation

3T3‐L1 adipocytes cultured in six‐well plates were exposed to EVs (30 μg/mL) derived from PC3 and DU145 cells for 48 h. After treatment, cells were collected, washed with PBS, and incubated with 1 μM Bodipy (Thermo Fisher Scientific) for 30 min. Flow cytometry was performed using a NovoCyte 3000 instrument (ACEA Biosciences, San Diego, CA), and data were analyzed with NovoExpress software.

PCa cells were seeded in six‐well plates at a density of 5 × 10^4^ cells/well. After 48 h, cells were exposed to conditioned medium (CM) collected from EV‐treated adipocytes for 24 h (while CM from untreated adipose cells was used for controls). Subsequent processing was performed as indicated above.

### 2.8 Measurement of FFA Release and Uptake

3T3L‐1 adipocytes cultured in 6‐well plates were exposed to EVs (30 μg/mL) from PC3 and DU145 cells for 48 h. FFA release was assessed using a FFA assay kit (Sigma‐Aldrich) and quantified by EnSpire Multimode Plate reader (PerkinElmer, Milano, Italy). In the case of FFA uptake, PCa cells were seeded in six‐well plates (5 × 10^4^ cells/well), and, after 48 h, they were exposed to CM from PCa EV‐treated adipocytes for 24 h (while CM from untreated adipose cells was used for controls). FFA levels were then measured in their medium as indicated above.

### 2.9 Measurement of Glycerol Release

3T3L‐1 adipocytes cultured in six‐well plates were exposed to EVs (30 μg/mL) from PC3 and DU145 cells for 48 h. Glycerol release was assessed using a glycerol assay kit (Abcam) and quantified by the EnSpire Multimode Plate reader.

### 2.10 Measurement of cAMP Levels

3T3L‐1 adipocytes cultured in six‐well plates were exposed to EVs (30 μg/mL) from PC3 and DU145 cells for 6 h. Cyclic AMP (cAMP) levels were assessed by using a cAMP parameter assay kit (R&D Systems, Minneapolis, MN, USA) and measured by an EnSpire Multimode Plate reader.

### 2.11 Cell Proliferation Assay

PCa cells were seeded in 6‐well plates (5 × 10^4^ cells/well). After 48 h (*t* = 0), they were exposed to CM from PCa EV‐treated adipocytes for 96 h (while CM from untreated adipose cells was used for controls). Cells were collected, mixed 1:1 (v/v) with 0.4% Trypan Blue solution, and subsequently counted using a Luna automated cell counter (Logos Biosystems, Annandale, VA, USA).

### 2.12 Colony Formation Assay

PCa cells were seeded in six‐well plates (100–250 cells/well, depending on the cell type). After 48 h, cells were exposed to CM from PCa EV‐treated adipocytes for 72 h (while CM from untreated adipose cells was used for controls), followed by culture in complete medium for an additional 7–10 days to allow colony formation. Colonies were fixed with 70% methanol and stained with Crystal Violet 0.15%.

### 2.13 Wound Healing Assay

PCa cells were seeded in six‐well plates (2 × 10^5^ cells/well). After 48 h (*t* = 0), a wound healing assay was performed by scratching the confluent cell monolayer, followed by treatment with CM from EV‐treated adipocytes (while CM from untreated adipose cells was used for controls); mitomycin C (5 μg/mL) was used to prevent cell proliferation. Images of the wound area were captured immediately after scratching (0 h) and at 12 and 24 h using a Zeiss Axiovert 200 microscope equipped with a 63×/1.4 objective lens and a CoolSNAP ES CCD camera (Roper Scientific–Crisel Instruments, Rome, Italy).

### 2.14 Migration Assay

PCa cells were seeded in six‐well plates (5 × 10^4^ cells/well); after 48 h, they were exposed to CM from PCa EV‐treated adipocytes for 24 h (while CM from untreated adipose cells was used for controls). Subsequently, cells were collected and seeded into the upper chamber of a transwell 24‐well plate (1 × 10^5^ cells/well) containing an 8‐μm pore membrane. Complete growth medium was added to the lower chamber as a chemoattractant. After 6 h of incubation, migrated cells on the lower surface of the membrane were fixed, stained with a Diff‐Quick staining kit (DADE, Dudingen, Switzerland) and counted.

### 2.15 Anoikis Assay

PCa cells were seeded in ultra‐low attachment 24‐well plates (5 × 10^4^ cells/well) and cultured in CM from PCa EV‐treated adipocytes for 48 h (while CM from untreated adipose cells was used for controls). Cells were collected, mixed 1:1 (v/v) with 0.4% Trypan Blue solution, and subsequently counted using a Luna automated cell counter.

### 2.16 Invasion Assay

PCa cells were seeded in six‐well plates (5 × 10^4^ cells/well); after 48 h, they were exposed to CM from PCa EV‐treated adipocytes for 24 h (while CM from untreated adipose cells was used for controls). Subsequently, cells were collected and seeded into the upper chamber of a transwell 24‐well plate (1 × 10^5^ cells/well) containing a Matrigel‐coated 8‐μm pore membrane. Complete growth medium was added to the lower chamber as a chemoattractant. After 12 h of incubation, invaded cells on the lower surface of the membrane were fixed, stained with Diff‐Quick staining kit and counted.

### 2.17 Delipidation of Adipocyte Medium

Lipid depletion was performed by adding fumed silica (Sigma‐Aldrich) to CM from PCa EV‐treated adipocytes (20 mg/mL), followed by mixing overnight, centrifugation (2000 *g* for 15 min) and sterile filtration.

### 2.18 Akt Pharmacological Inhibition

Akt inhibition was achieved by treating PCa cells with BEZ235 (100 nM) for 24–72 h (depending on the assay), following exposure to CM from PCa EV‐treated adipocytes for 3 h (while CM from untreated adipose cells was used for controls).

### 2.19 Western Blot Analysis

Adipose and PCa cells and EVs were lysed using RIPA buffer. Twenty micrograms of proteins per sample were separated by SDS‐PAGE and transferred onto nitrocellulose membranes. Membranes were probed with specific primary antibodies, followed by HRP‐conjugated secondary antibodies. Protein bands were detected using ECL (Westar Etac Ultra 2.0, XLS075,0100, Cyanagen). GAPDH was used as a loading control.

### 2.20 Statistical Analysis

Statistical analyses were performed using GraphPad Prism 5 (GraphPad Software, San Diego, CA, USA). Data are presented as mean ± SEM from three independent experiments. Differences between groups were evaluated using either a *t*‐test or one‐way analysis of variance (ANOVA) followed by Dunnett's or Bonferroni's *post hoc* test. A *p*‐value of less than 0.05 was considered statistically significant.

## 3 Results

### 3.1 Characterization of PCa EVs

**FIGURE 1:**
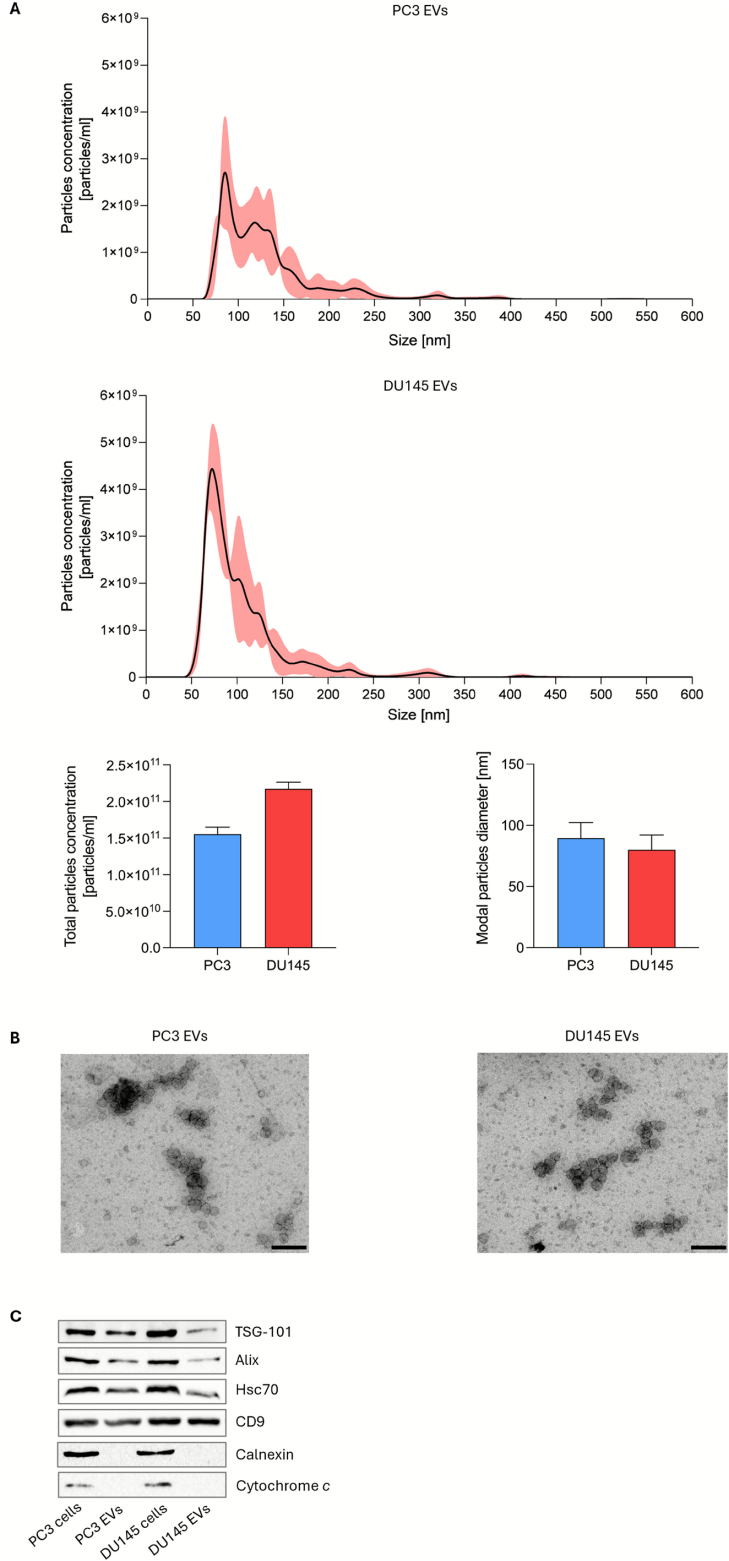
Characterization of PCa EVs. (A) EV size distribution measured by nanoparticle tracking analysis. (B) EV morphology visualized by transmission electron microscopy. Scale bars are 300 nm. (C) Western blot analysis was performed to investigate the expression levels of TSG101, Alix, Hsc70, CD9, calnexin, and cytochrome *c* in PC3 and DU145 cells and in their EVs. One representative of three experiments performed is shown.

Before determining the role of EVs in the PCa microenvironment, we characterized the tumor‐derived particles. Vesicles were isolated from PC3 and DU145 cell CM by SEC, and they were shown to exhibit an average size of 50–160 nm (Figure 1A) and a round morphology (Figure 1B). Moreover, they displayed different exosomal hallmarks, such as TSG101, Alix, Hsc70, and CD9, without cellular contaminants, as suggested by the lack of calnexin and cytochrome *c* (Figure 1C). Collectively, these data prove that our purified samples specifically contain small EVs.

### 3.2 PCa EVs Promote Lipolysis in Adipocytes

**FIGURE 2:**
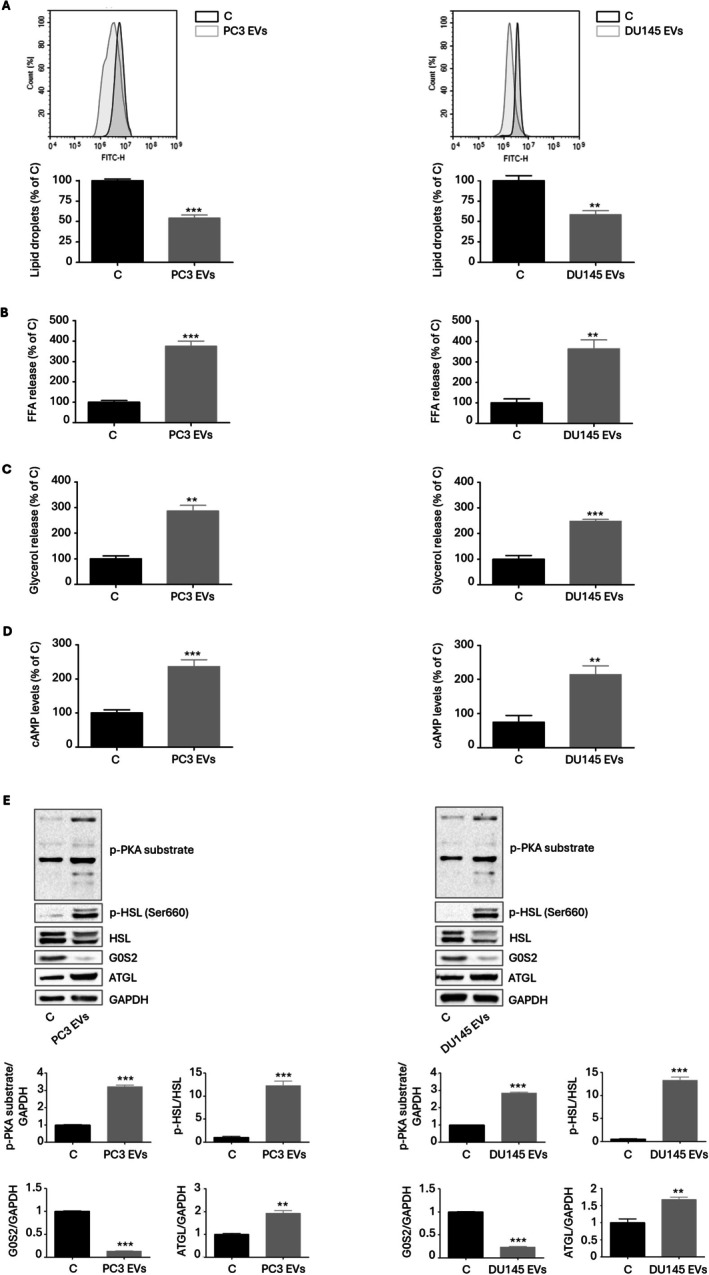
PCa EVs promote lipolysis in adipocytes. (A) 3T3‐L1 adipocytes were incubated with PC3 and DU145 EVs (30 μg/mL) for 48 h. Lipid accumulation was then evaluated by cytofluorimetric analysis after staining with Bodipy 1 μM for 30 min. Each experiment was repeated three times. Data represent mean values ± SEM and were analyzed by *t*‐test. **p < 0.01 vs. C (control), ***p < 0.001 vs. C (control). (B) 3T3‐L1 adipocytes were incubated with PC3 and DU145 EVs (30 μg/mL) for 48 h. FFA release was then evaluated by colorimetric assay. Each experiment was repeated three times. Data represent mean values ± SEM and were analyzed by *t*‐test. **p < 0.01 vs. C (control), ***p < 0.001 vs. C (control). (C) 3T3‐L1 adipocytes were incubated with PC3 and DU145 EVs (30 μg/mL) for 48 h. Glycerol release was then evaluated by colorimetric assay. Each experiment was repeated three times. Data represent mean values ± SEM and were analyzed by *t*‐test. **p < 0.01 vs. C (control), ***p < 0.001 vs. C (control). (D) 3T3‐L1 adipocytes were incubated with PC3 and DU145 EVs (30 μg/mL) for 6 h. cAMP levels were then evaluated by ELISA assay. Each experiment was repeated three times. Data represent mean values ± SEM and were analyzed by *t*‐test. **p < 0.01 vs. C (control), ***p < 0.001 vs. C (control). (E) After PCa EV treatment (30 μg/mL for 12 h), Western blot analysis was performed to investigate the expression levels of PKA substrates, HSL, G0S2, and ATGL in 3T3‐L1 adipocytes. GAPDH expression was evaluated as a loading control. One representative of three experiments performed is shown. Data represent mean values ± SEM and were analyzed by *t*‐test. **p < 0.01 vs. C (control), ***p < 0.001 vs. C (control).

We next hypothesized that PCa EVs could affect the behavior of adipocytes, driving their switch toward a cancer‐associated metabolic phenotype. To verify this hypothesis, mature 3T3‐L1 cells were treated with vesicles from PC3 and DU145 cell lines at a concentration of 30 μg/mL, a dose mirroring the physiologically relevant EV levels in the tumor microenvironment that is typically used for functional assays in the oncology field [18, 19, 20, 21, 22]. Interestingly, this resulted in the stimulation of lipolysis, with reduced intracellular lipid accumulation and enhanced FFA and glycerol release (Figure 2A–C). Mechanistically, tumor‐released particles induced an increase in the levels of cAMP, followed by the activation of protein kinase A (PKA) (as indicated by the phosphorylation of its substrates) and hormone‐sensitive lipase (HSL); parallelly, inhibition of G0/G1 switch protein 2 (G0S2) and upregulation of adipose triglyceride lipase (ATGL) were observed (Figure 2D,E). In this setting, it should be emphasized that the vesicles isolated from the medium of RWPE‐1 normal prostate epithelial cells did not affect adipocyte phenotype (Figure S1). Taken together, these findings indicate that the EVs obtained from PCa cells can elicit the lipolytic process in adipose cells, conferring cancer‐associated traits to the latter.

### 3.3 The Secretome From EV‐Treated Adipocytes Increases PCa Cell Growth

**FIGURE 3:**
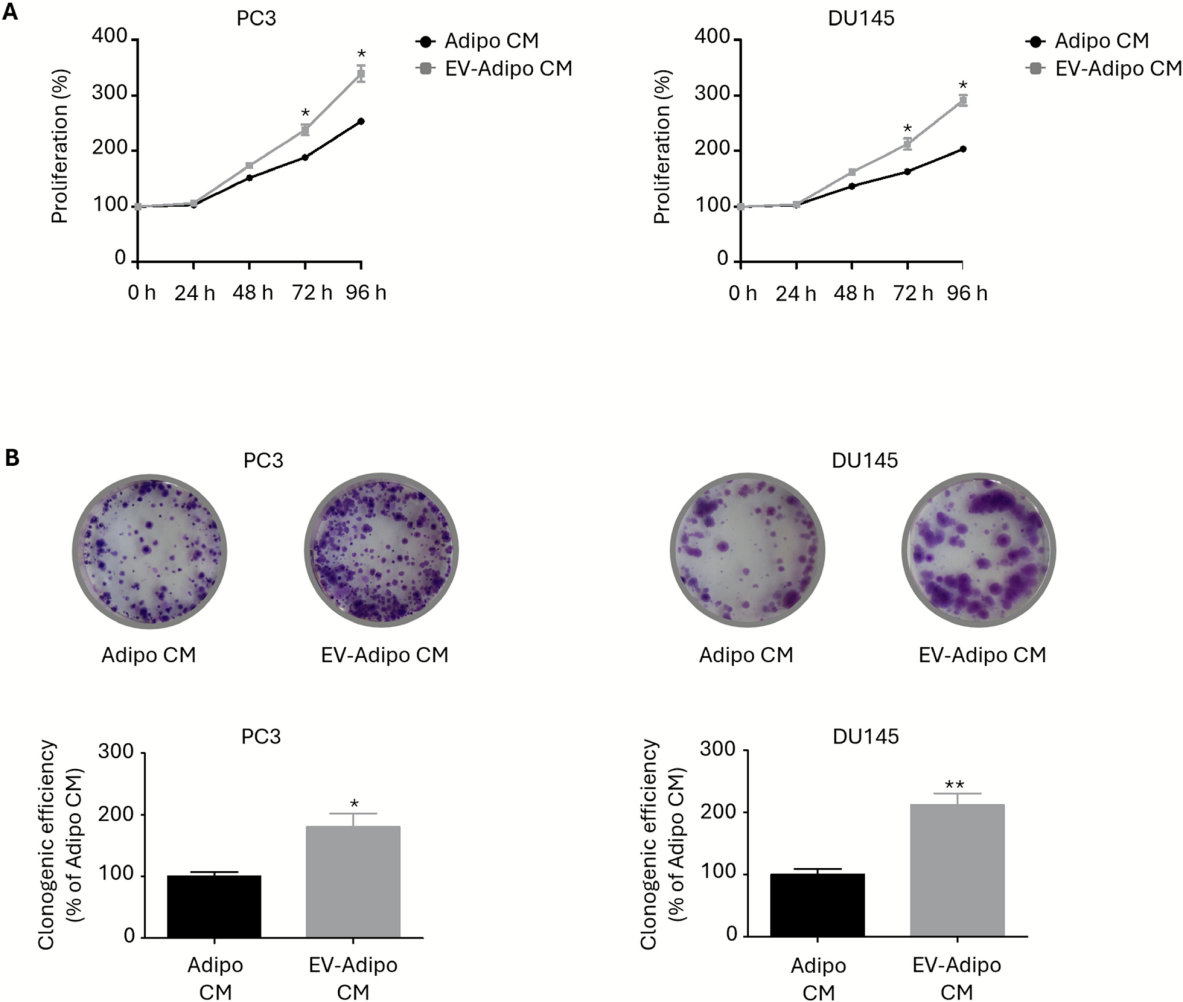
The secretome from EV‐treated adipocytes increases PCa cell growth. (A) 3T3‐L1 adipocytes were incubated with PC3 and DU145 EVs (30 μg/mL) for 48 h, and their medium was then given to PC3 and DU145 cells (96 h). Cell proliferation was then evaluated by Trypan Blue exclusion assay. Each experiment was repeated three times. Data represent mean values ± SEM and were analyzed by *t*‐test. *p < 0.05 vs. Adipo CM (control). (B) 3T3‐L1 adipocytes were incubated with PC3 and DU145 EVs (30 μg/mL) for 48 h, and their medium was then given to PC3 and DU145 cells (72 h). Clonogenic ability was then evaluated by colony formation assay. Each experiment was repeated three times. Data represent mean values ± SEM and were analyzed by *t*‐test. *p < 0.05 vs. Adipo CM (control), **p < 0.01 vs. Adipo CM (control).

To investigate the role of adipose tissue in mediating PCa aggressiveness, adipocytes were incubated with PC3 and DU145 cell‐derived EVs, and their CM (EV‐adipo CM) was used to treat both PCa cell lines. Indeed, while our group and others have already clarified the pro‐tumor activity of the adipocyte secretome per se [17, 23, 24, 25, 26], the specific influence of tumor‐derived vesicles on the composition and function of these secreted factors remains to be determined. As illustrated in Figure 3A, EV‐adipo CM significantly enhanced cancer cell proliferation. In addition, it significantly increased tumor clonogenic ability, leading to a 1.8‐ and 2.1‐fold increase in the colony formation efficiency of PC3 and DU145 cells, respectively (Figure 3B). Overall, these observations suggest that the secretome from EV‐treated adipocytes can stimulate PCa cell growth.

### 3.4 The Secretome From EV‐Exposed Adipocytes Enhances PCa Cell Invasive Potential

**FIGURE 4:**
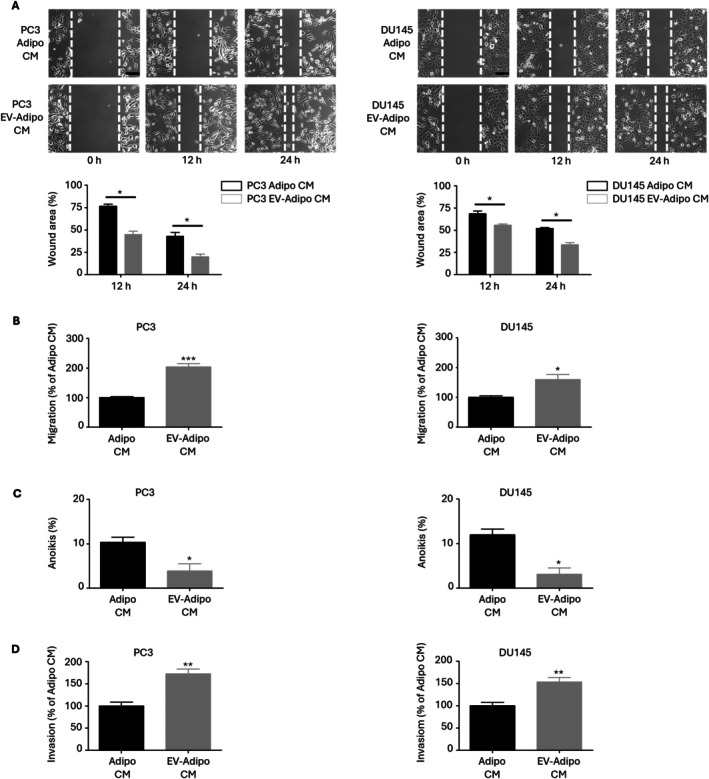
The secretome from EV‐exposed adipocytes enhances PCa cell invasive potential. (A) 3T3‐L1 adipocytes were incubated with PC3 and DU145 EVs (30 μg/mL) for 48 h, and their medium was then given to PC3 and DU145 cells (24 h). Cell migration was then evaluated by wound healing assay. Each experiment was repeated three times. Data represent mean values ± SEM and were analyzed by *t*‐test. *p < 0.05. Scale bars are 200 μm. (B) 3T3‐L1 adipocytes were incubated with PC3 and DU145 EVs (30 μg/mL) for 48 h, and their medium was then given to PC3 and DU145 cells (24 h). Cell migration was then evaluated by transwell assay. Each experiment was repeated three times. Data represent mean values ± SEM and were analyzed by *t*‐test. *p < 0.05 vs. Adipo CM (control), ***p < 0.001 vs. Adipo CM (control). (C) 3T3‐L1 adipocytes were incubated with PC3 and DU145 EVs (30 μg/mL) for 48 h, and their medium was then given to PC3 and DU145 cells (48 h). Anoikis was then evaluated by Trypan Blue exclusion assay. Each experiment was repeated three times. Data represent mean values ± SEM and were analyzed by *t*‐test. *p < 0.05 vs. Adipo CM (control). (D) 3T3‐L1 adipocytes were incubated with PC3 and DU145 EVs (30 μg/mL) for 48 h, and their medium was then given to PC3 and DU145 cells (24 h). Cell invasion was then evaluated by transwell assay. Each experiment was repeated three times. Data represent mean values ± SEM and were analyzed by *t*‐test. **p < 0.01 vs. Adipo CM (control).

To further assess the effects of the secretome from EV‐exposed adipocytes on PCa malignant traits, we focused our attention on tumor cell invasive potential. Intriguingly, we found that EV‐adipo CM could boost PCa cell migration, almost doubling it in PC3 cells, while determining a 1.6‐fold increase in DU145 cells (Figure 4A,B). Furthermore, it could prevent the induction of anoikis, a cell death process representing a crucial metastasis‐limiting step as it occurs after disruption of the integrin‐mediated interactions with the extracellular matrix (ECM) (Figure 4C). Remarkably, this resulted in enhanced cell invasion (Figure 4D). Once again, these results highlight the pivotal function exerted by EV‐treated adipocytes in the control of PCa malignancy.

### 3.5 FFAs in EV‐Conditioned Adipocyte Secretome Are Taken Up by PCa Cells and Mediate the Above Pro‐Tumor Effects

**FIGURE 5:**
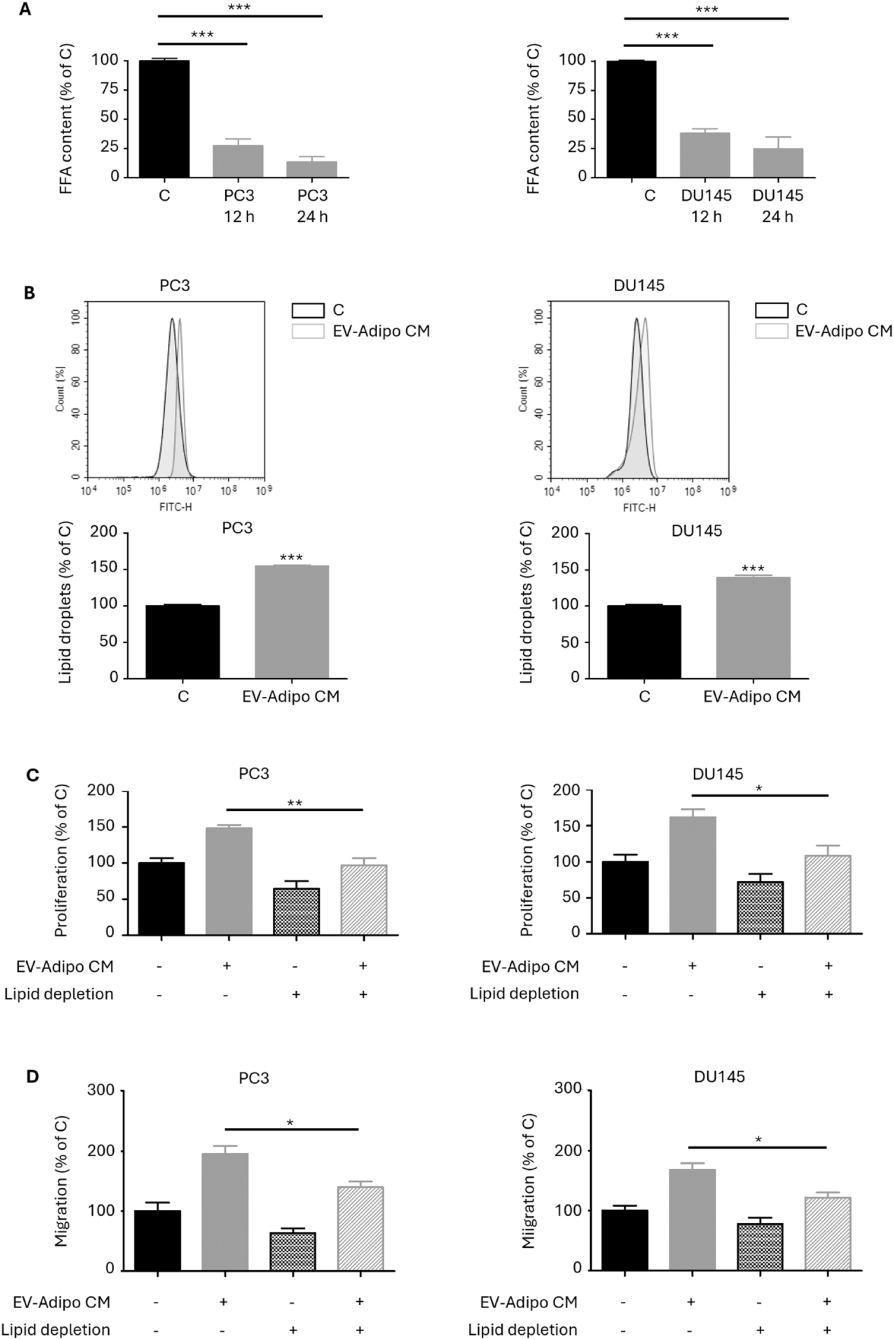
FFAs in EV‐conditioned adipocyte secretome are taken up by PCa cells and mediate the above pro‐tumor effects. (A) 3T3‐L1 adipocytes were incubated with PC3 and DU145 EVs (30 μg/mL) for 48 h, and their medium was then given to PC3 and DU145 cells (24 h). FFA uptake was then evaluated by colorimetric assay. Each experiment was repeated three times. Data represent mean values ± SEM and were analyzed by one‐way ANOVA followed by Dunnet's test. ***p < 0.001. (B) 3T3‐L1 adipocytes were incubated with PC3 and DU145 EVs (30 μg/mL) for 48 h, and their medium was then given to PC3 and DU145 cells (24 h). Lipid accumulation was then evaluated by cytofluorimetric analysis after staining with Bodipy 1 μM for 30 min. Each experiment was repeated three times. Data represent mean values ± SEM and were analyzed by *t*‐test. ***p < 0.001 vs. C (control). (C) After delipidation, EV‐Adipo CM was given to PC3 and DU145 cells (72 h). Cell proliferation was then evaluated by Trypan Blue exclusion assay. Each experiment was repeated three times. Data represent mean values ± SEM and were analyzed by one‐way ANOVA followed by Bonferroni's test. *p < 0.05, **p < 0.01. (D) After delipidation, EV‐Adipo CM was given to PC3 and DU145 cells (24 h). Cell migration was then evaluated by transwell assay. Each experiment was repeated three times. Data represent mean values ± SEM and were analyzed by one‐way ANOVA followed by Bonferroni's test. *p < 0.05.

Then, we evaluated the contribution of the FFAs released from EV‐conditioned adipocytes to the phenotypic changes observed in PCa cells. First, we found that the FFAs contained in EV‐adipo CM were taken up by PCa cells, as indicated by the time‐dependent decrease in their levels when given to tumor cells as well as by the subsequent accumulation of lipid droplets in PC3 and DU145 cells themselves (Figure 5A,B). More importantly, lipid depletion significantly reduced the ability of this medium to stimulate PCa cell growth and migration (Figure 5C,D). Based on these data, we can conclude that the vesicles from PCa cells can effectively promote the release of FFAs from adipocytes, which in turn sustain tumor cell aggressiveness.

### 3.6 FFAs in EV‐Conditioned Adipocyte Secretome Trigger the Akt Signaling in PCa Cells

**FIGURE 6:**
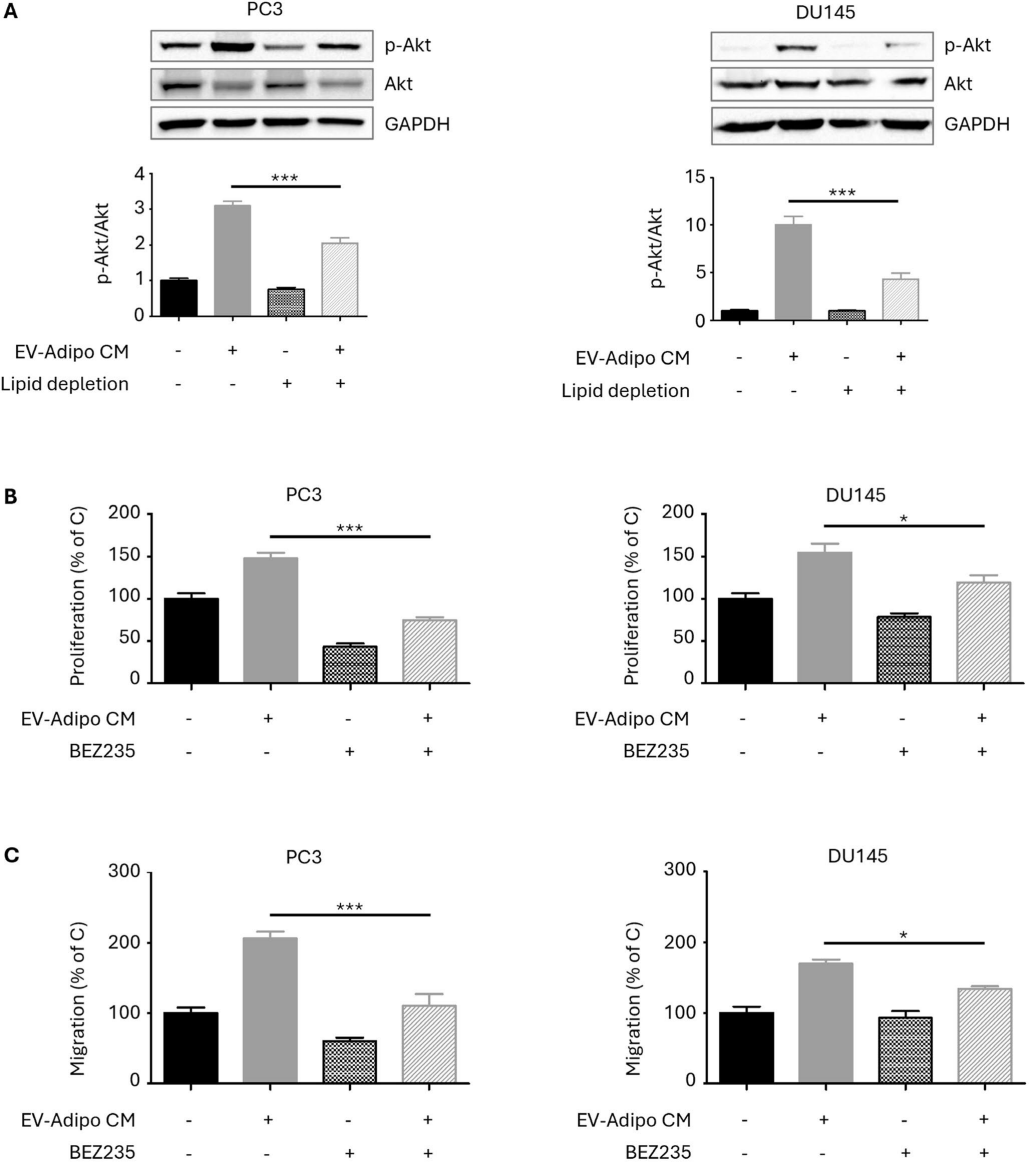
FFAs in EV‐conditioned adipocyte secretome trigger the Akt signaling in PCa cells. (A) After EV‐Adipo CM treatment (24 h), Western blot analysis was performed to investigate the expression levels of p‐Akt in PC3 and DU145 cells. GAPDH expression was evaluated as a loading control. One representative of three experiments performed is shown. Data represent mean values ± SEM and were analyzed by one‐way ANOVA followed by Bonferroni's test. ***p < 0.001 vs. C (control). (B) After BEZ235 treatment (100 nM, 72 h) following EV‐Adipo CM treatment (3 h), PC3 and DU145 cell proliferation was evaluated by Trypan Blue exclusion assay. Each experiment was repeated three times. Data represent mean values ± SEM and were analyzed by one‐way ANOVA followed by Bonferroni's test. *p < 0.05, ***p < 0.001. (C) After BEZ235 treatment (100 nM, 24 h) following EV‐Adipo CM treatment (3 h), PC3 and DU145 cell migration was evaluated by transwell assay. Each experiment was repeated three times. Data represent mean values ± SEM and were analyzed by one‐way ANOVA followed by Bonferroni's test. *p < 0.05, ***p < 0.001.

We finally dissected the molecular mechanisms by which EV‐exposed adipocytes could modulate PCa cell malignancy. As shown in Figure 6A, phosphorylation of the Akt pathway was observed not only in PI3K‐overexpressing PC3 cells but also in PI3K‐wild type DU145 cells treated with EV‐adipo CM. Of note, the induction of this cascade was abrogated upon EV‐adipo CM delipidation, indicating that the FFAs contained in adipocyte secretome are crucial activators of the above molecular signature. Of course, this has important therapeutic implications. Indeed, the above molecular vulnerabilities could be exploited for the design of new and more effective anti‐PCa approaches. In this setting, the anti‐cancer effects of BEZ235, a novel PI3K inhibitor, were tested on PCa cells. Figure 6B,C show that treatment of tumor cell lines with this drug significantly counteracted the pro‐tumor activity of EV‐adipo CM, demonstrating that the crosstalk between adipose cells and PCa can be successfully suppressed by targeting the Akt signaling.

## 4 Discussion

Obesity not only represents a major health problem per se, but it has been recently associated with the development of several malignancies, including lethal PCa. Indeed, obese individuals display a higher risk of PCa progression and recurrence compared to men of normal weight [5, 6, 7]. In addition, increased periprostatic adipose tissue density is linked to a more aggressive form of the disease [10, 23]. Specifically, the dysfunctional adipose mass in obese patients is a preeminent source of metabolites, which are deeply involved in tumorigenesis [5, 6, 7, 10]. On the contrary, the role of EVs in PCa evolution is still poorly understood.

In this study, we examined how EVs from PCa cells influence the phenotype of 3T3‐L1 adipocytes to elucidate their role in modulating the communication between these cell types.

First, we characterized the EVs derived from PC3 and DU145 cells, showing that they displayed a mean diameter of about 100 nm and a spherical shape. In addition, they were found to contain different vesicular markers, including TSG101, Alix, HSC70, and CD9. Overall, these observations allowed us to conclude that our samples were enriched in small vesicles, as suggested by the “minimal information for studies of extracellular vesicles 2023” (MISEV2023) guidelines [27].

Then, we demonstrated that the EVs released from PCa cells triggered lipolysis in adipocytes, which exhibited decreased lipid content and enhanced FFA and glycerol secretion; in particular, these changes were accompanied by the inhibition of G0S2, upregulation of ATGL and activation of the cAMP/PKA/HSL signaling pathway. This is a typical hallmark of cancer‐associated adipocytes (CAAs), which are known to accumulate a reduced number of cytoplasmic triglycerides [28, 29]. Remarkably, our data align with recent reports emphasizing the capability of EVs from different tumor types, including breast, colon, liver, lung, and pancreatic cancer, to induce the release of FFAs from neighboring adipose cells [14, 15, 16].

A common characteristic of CAAs is the acquisition of a tumor‐supporting phenotype [28, 29]. In this regard, we observed that treatment of PCa cells with EV‐adipo CM led to a significant increase in cancer cell proliferation and colony formation efficiency. Moreover, it resulted in higher migration and resistance to anoikis, eventually culminating in improved invasion. These results are in agreement with prior studies evidencing the critical role of breast‐ and hepatocarcinoma‐secreted particles in the pro‐tumor transformation of adipose cells [14, 15]. Our findings not only corroborate the capacity of PCa cells to directly educate adipocytes into CAAs but also open up the possibility that their EVs are deeply implicated in the emergence of a tumor‐sustaining microenvironment.

As a further step in determining the cruciality of the FFAs secreted by EV‐treated adipocytes in PCa aggressiveness, we analyzed their uptake kinetics in tumor cells, showing that about 75% of them are actively internalized after 24 h, leading to an increased lipid droplet formation; on the other hand, delipidation of the CM from EV‐conditioned adipose cells significantly affected its tumor‐sustaining ability. In particular, the FFAs contained in EV‐Adipo CM were observed to activate the Akt signaling in cancer cells. Accordingly, adipocyte‐derived lipids have been deeply implicated in the development of a variety of malignancies, including metastatic and taxane‐insensitive PCa [28, 29]. On the other hand, it should be emphasized that adipocytes can exert paracrine effects on numerous carcinomas by boosting PI3K‐related tumor growth and survival [30, 31, 32, 33, 34, 35]. To our knowledge, this is the first study pointing out the involvement of the above tumor‐promoting cascade in the dialog between adipose mass and PCa.

Given the above findings, we finally validated Akt phosphorylation as a target to disrupt the adipose‐to‐PCa cell communication. Indeed, although drugs targeting this protein have demonstrated significant efficacy in directly managing tumor cells [36, 37], their effects within the context of the interactions between malignant cells and adipocytes remain unexplored. Herein, we found that treatment of PC3 and DU145 cell lines with the PI3K inhibitor BEZ235 successfully abolished the pro‐tumor effects of adipocytes, further supporting the potential role of Akt signaling in promoting the obesity‐mediated PCa evolution, as previously highlighted in melanoma and colorectal, ovarian and breast carcinoma [34, 38, 39]. Taken together, our observations validate the hypothesis that PCa vesicles can educate adipose cells toward a tumor‐supporting state able to drive cancer malignancy through the release of FFAs and further indicate that molecular targeting of Akt might be exploited to prevent cancer progression.

In conclusion, this study provides novel insights into the mechanisms governing the interplay between PCa and adipose tissue, revealing that cancer‐released EVs can endow adipocytes with pro‐tumor properties by stimulating lipolysis. This may lead to the assumption that an EV‐coordinated bidirectional communication exists between the tumor and adipose mass, presumably contributing to the poor cancer prognosis occurring in the case of an obese condition. Since our findings are based solely on in vitro experiments, in vivo studies will be essential to validate and expand upon these results. Taken together, our data may lay the groundwork for future investigations into the role of adipose tissue in shaping the PCa microenvironment.

## Author Contributions

Study design: Fabrizio Fontana. Data collection: Gaia Giannitti, Kinga Kamińska, Sara Marchesi, Riccardo Garavaglia, Ivan Preosto, and Fabrizio Fontana. Data analysis: Gaia Giannitti, Kinga Kamińska, Sara Marchesi, Riccardo Garavaglia, Ivan Preosto, and Fabrizio Fontana. Manuscript preparation: Kinga Kamińska, Małgorzata Grzesiak, and Fabrizio Fontana. Funding acquisition: Fabrizio Fontana.

## Funding

This research was funded by MIUR Progetto di Eccellenza (Department of Pharmacological and Biomolecular Sciences “Rodolfo Paoletti”, Università degli Studi di Milano). F.F. was supported by an AIRC Fellowship for Italy (from April 1, 2020 to March 31, 2023).

## Conflicts of Interest

The authors declare no conflicts of interest.

## Supporting information


**Figure S1:** EVs from normal prostate epithelial cells do not affect adipocyte phenotype. (A) 3T3‐L1 adipocytes were incubated with RWPE‐1 EVs (30 μg/mL) for 48 h. Lipid accumulation was then evaluated by cytofluorimetric analysis after staining with Bodipy 1 μM for 30 min. Each experiment was repeated three times. Data represent mean values ± SEM and were analyzed by *t*‐test. (B) 3T3‐L1 adipocytes were incubated with RWPE‐1 EVs (30 μg/mL) for 48 h. FFA release was then evaluated by colorimetric assay. Each experiment was repeated three times. Data represent mean values ± SEM and were analyzed by *t*‐test. (C) 3T3‐L1 adipocytes were incubated with RWPE‐1 EVs (30 μg/mL) for 48 h. Glycerol release was then evaluated by colorimetric assay. Each experiment was repeated three times. Data represent mean values ± SEM and were analyzed by *t*‐test.

## Data Availability

The data that support the findings of this study are available from the corresponding author upon reasonable request.
