# Supplementary figures and images for: Extracellular Vesicles Released From Prostate Cancer Cells Confer Pro‐Tumor Properties to Adipocytes by Stimulating Lipolysis
Source: Biofactors. 2025 Dec 11;51(6):e70067. doi: 10.1002/biof.70067 (PMC12699169; doi:10.1002/biof.70067)

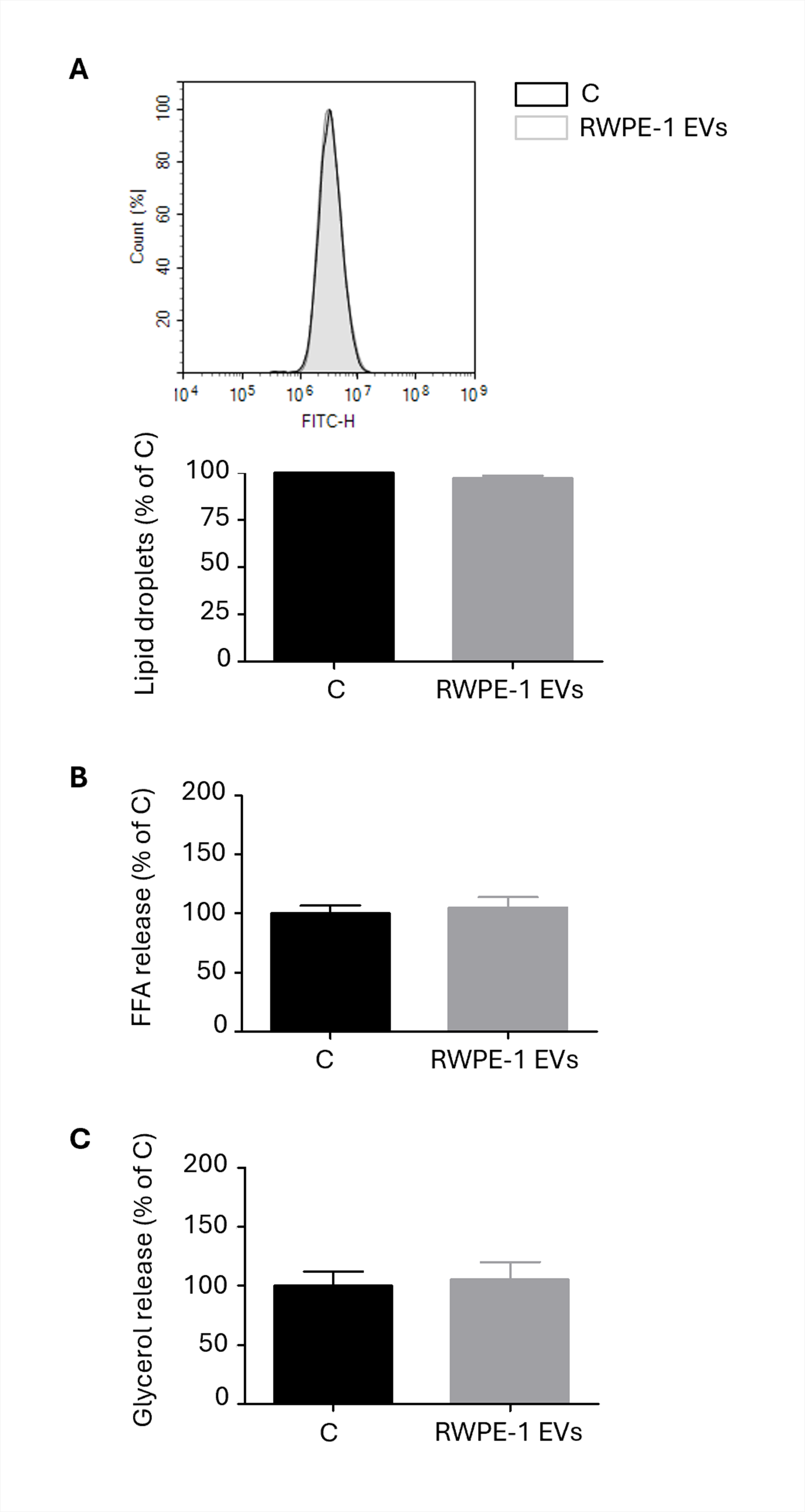

Supplement: Supplementary file 1 — Figure S1: EVs from normal prostate epithelial cells do not affect adipocyte phenotype. (A) 3T3‐L1 adipocytes were incubated with RWPE‐1 EVs (30 μg/mL) for 48 h. Lipid accumulation was then evaluated by cytofluorimetric analysis after staining with Bodipy 1 μM for 30 min. Each experiment was repeated three times. Data represent mean values ± SEM and were analyzed by t‐test. (B) 3T3‐L1 adipocytes were incubated with RWPE‐1 EVs (30 μg/mL) for 48 h. FFA release was then evaluated by colorimetric assay. Each experiment was repeated three times. Data represent mean values ± SEM and were analyzed by t‐test. (C) 3T3‐L1 adipocytes were incubated with RWPE‐1 EVs (30 μg/mL) for 48 h. Glycerol release was then evaluated by colorimetric assay. Each experiment was repeated three times. Data represent mean values ± SEM and were analyzed by t‐test. [file BIOF-51-0-s001.tif]
